# An economic appraisal of the Australian Medical Sheepskin for the prevention of sacral pressure ulcers from a nursing home perspective

**DOI:** 10.1186/1472-6963-10-226

**Published:** 2010-08-05

**Authors:** Patriek Mistiaen, Andre Ament, Anneke L Francke, Wilco Achterberg, Ruud Halfens, Janneke Huizinga, Henri Post

**Affiliations:** 1 NIVEL, Netherlands Institute for Health Services Research, PO Box 1568, 3500 BN Utrecht, The Netherlands; 2 Maastricht University, Department of Health Organisation Policy and Economics, PO Box 616, 6200 MD Maastricht, the Netherlands; 3 EMGO Institute for Health and Care Research (EMGO+) of the VU University Medical Center Amsterdam, Department of Public and Occupational Health, van de Boechorststraat 7, 1081 ET Amsterdam, the Netherlands; 4 EMGO Institute for Health and Care Research (EMGO+) of the VU University Medical Center Amsterdam, Department of Nursing Home Medicine, van de Boechorststraat 7, 1081 ET Amsterdam, The Netherlands; 5 Maastricht University, Faculty of Health, Medicine and Life Sciences, PO Box 616, 6200 MD Maastricht, the Netherlands; 6 Witten-Herdecke University, Fakultät für Medizin, Institut für Pflegewissenschaft, Stockumer Straße 12, 58453 Witten, Germany; 7 V&VN- Dermatology, National Nursing and Caring Organisation, department for dermatology nursing, PO Box 8212, 3503 RE Utrecht, the Netherlands; 8 Evean Zorg, Bristolroodstraat 164, 1503 NZ Zaandam, the Netherlands

## Abstract

**Background:**

Many devices are in use to prevent pressure ulcers, but from most little is known about their effects and costs. One such preventive device is the Australian Medical Sheepskin that has been proven effective in three randomized trials. In this study the costs and savings from the use of the Australian Medical Sheepskin were investigated from the perspective of a nursing home.

**Methods:**

An economic model was developed in which monetary costs and monetary savings in respect of the sheepskin were balanced against each other. The model was applied to a fictional (Dutch) nursing home with 100 beds for rehabilitation patients and a time horizon of one year. Input variables for the model consisted of investment costs for using the sheepskin (purchase and laundry), and savings through the prevented cases of pressure ulcers. The input values for the investment costs and for the effectiveness were empirically based on a trial with newly admitted rehabilitation patients from eight nursing homes. The input values for the costs of pressure ulcer treatment were estimated by means of four different approaches.

**Results:**

Investment costs for using the Australian Medical Sheepskin were larger than the monetary savings obtained by preventing pressure ulcers. Use of the Australian Medical Sheepskin involves an additional cost of approximately €2 per patient per day. Preventing one case of a sacral pressure ulcer by means of the Australian Medical Sheepskin involves an investment of €2,974 when the sheepskin is given to all patients. When the sheepskin is selectively used for more critical patients only, the investment to prevent one case of sacral pressure ulcers decreases to €2,479 (pressure ulcer risk patients) or €1,847 (ADL-severely impaired patients). The factors with the strongest influence on the balance are the frequency of changing the sheepskin and the costs of washing related to this. The economic model was hampered by considerable uncertainty in the estimations of the costs of pressure ulcer treatment.

**Conclusions:**

From a nursing home perspective, the investment costs for use of the Australian Medical Sheepskin in newly admitted rehabilitation patients are larger than the monetary savings obtained by preventing pressure ulcers.

## Background

Pressure ulcers are a highly prevalent problem, especially in nursing home patients [1,2]. They have a large impact on quality of life [3,4] and high costs are involved in the treatment [5-7].

However, pressure ulcers can to a large extent be prevented by adequate care. There are many devices to help in the prevention of pressure ulcers [8], but for many there is lack of knowledge about the (cost)-effectiveness [9,10]. However, governments increasingly urge guideline developers to base their recommendations regarding the use of certain devices not only on effectiveness data but also on economic considerations.

One such pressure ulcer preventing device is the Australian Medical Sheepskin (AMS), that has been proven effective in two RCTs in hospital patients [11,12] and in a recent RCT in a nursing home setting [13,14].

The Australian Medical Sheepskin is a real genuine sheepskin, coming from the Australian Merino sheep. The sheepskin has a wool pile length of 30 mm, and a high density of wool piles, and reduces pressure and friction making it a possible good intervention for the prevention of pressure ulcers. The sheepskin is tanned and processed in such a way that it has an increased resistance to urine and can withstand up to 100 washes at 80°C to achieve high-level thermal disinfection, without losing its moisture absorbing and pressure relieving properties.

Although there are some studies [5,7,15-18] on the cost-effectiveness of preventative measures and appliances for pressure ulcers, no study known to us presents economical data for this Australian Medical Sheepskin.

In conjunction with the nursing home trial [13,14] economic data were gathered and will be presented in this article. The following research questions will be addressed:

1. What are the (yearly) investment costs for using the Australian Medical Sheepskin?

2. What are the (yearly) savings from use of the Australian Medical Sheepskin by preventing cases of pressure ulcers?

3. What is the balance between costs and savings from the use of the Australian Medical Sheepskin and what factors are most influential in this regard?

These questions will be answered through a cost-effectiveness analysis performed from the perspective of a fictional (Dutch) nursing home.

## Methods

The design for this study is a cost-effectiveness analysis, done in a model from the perspective of a fictional Dutch nursing home and with a time horizon of 1 year.

The pricing year was 2008 and actual prizes were applied so discounting was not needed, except for one estimate derived from a literature source. The currency used is Euro (€).

The research questions can be reworded as an equation: (Investment costs AMS (=purchase + laundry)) - (SAVINGS (=effectiveness AMS * costs of pressure ulcer treatment)) = BALANCE. Investment costs consist of costs related to purchase the AMS and costs related to using and washing the AMS. The savings are the number of patients that are prevented from a pressure ulcer by use of the AMS multiplied by the costs of treating a pressure ulcer. The equation was applied in a model for a fictional Dutch nursing home; the input values for the equation were based as much as possible on the empirical data from the above-mentioned RCT in eight Dutch nursing homes, and where needed, were supplemented through other sources. This trial formed the main source of data for the analysis of investments costs and the effectiveness. In the trial 588 newly admitted patients, mainly with a rehabilitation purpose, were randomized to either the control group that received usual care only or to the intervention group that received usual care *and *an Australian Medical Sheepskin. All patients were monitored for the development of sacral pressure ulcers from admission until 30 days thereafter. The experimental group received usual care plus an Australian Medical Sheepskin as a layer on the mattress within ultimately 48 hours after admission; the control group received usual care only. Usual care was all care that nursing wards normally applied for pressure ulcer prevention without any further standardization for this study. The incidence of sacral pressure ulcers grade 1 or higher was significantly lower in the experimental group than in the control group (8.9% versus 14.7%). This means a relative risk of 0.61 (95% CI: 0.37 to 0.96). These results are in line with the results of two earlier trials [11,12] on the Australian Medical Sheepskin in hospital patients.

Because only a few patients in that Dutch trial developed a sacral pressure ulcer, limiting the base for recording therapy costs, additional sources were used to estimate the pressure ulcer treatment costs.

All data obtained were then converted to input values for a fictional model nursing home anno 2008 for which yearly investment costs and savings were balanced.

### General model characteristics

The perspective for the analysis is the nursing home, meaning only expenses from and savings to the nursing home budget are taken into account. Only direct costs were counted. Assumed characteristics for the fictional nursing home of the model are that it has 100 beds for patients admitted for a primarily physical impairment, the occupancy rate is 100%, and it has 468 new admissions per year (based on a mean length of stay of 78 days as found in the Dutch trial [13]). The model was confined to direct monetary investments and monetary savings by the AMS use only. The time frame is one year.

Furthermore, the model is limited to savings from treatment of pressure ulcers in the sacral region only and solely grade 1 and 2. This choice is based on the fact that the primary outcome of the aforementioned trial was the incidence of sacral pressure ulcers and because sacral pressure ulcers are the most common form of pressure ulcers in nursing home patients. The limitation to grade 1 and 2 is based on the fact that grade 1 and 2 ulcers form the large majority (about 80%) of all sacral pressure ulcers in nursing homes [19-21] and there is no evidence yet that the AMS is also capable of preventing grade 3 or 4 pressure ulcers or of preventing pressure ulcers grade 1 or 2 from deteriorating to more severe grades. Furthermore in the model a ratio of 70% grade 1 versus 30% grade 2 pressure ulcers was used, as found in the Dutch trial.

In the model a base case was developed in which all variables were set to the most plausible values, based primarily on the trial and following discussion by the research project group that weighted additional sources. Thereafter one-way sensitivity analyses with a low and a high value for each of the input variables were performed to calculate the relative impact of the model components. In addition, the values for the sensitivity analyses were chosen after discussion in the research project group, based primarily on practical considerations rather than on mathematical grounds (e.g. the values for frequency of changing the AMS was varied between every 3 days to daily and every 5 days, instead of a percentage increase or decrease).

Since the monetary investments and the effectiveness depend on the number and kind of patients using the AMS, the model was calculated for 3 scenarios: AMS use for all patients, AMS use only for patients with a risk on pressure ulcers at admission (score ≤20 on the Braden-scale [22]) and AMS use only for patients that are severely ADL-impaired at admission (score <10 on the Barthel-index [23]). For the model the number of patients at risk for pressure ulcers (70%) and the number of patients that are severely ADL-impaired (45%) were derived from the Dutch trial [13].

Prices for products and devices used in the pressure ulcer treatment were based on the (Dutch) websites http://www.huidziekten.nl, or http://www.medicijnkosten.nl (these sites were visited during summer 2009). If no prices could be found at these sites data were used from a price list from a Dutch nursing home that participated in the trial. All prices were calculated on the basis of daily costs, accounting for the frequency of dressing change. Costs relating to salaries for personnel were based on actual gross mid-salary scales 2008 http://www.actiz.nl/cms/streambin.aspx?requestid=3517B57E-F9F5-4D8D-A682-1E92DAABFA50[24] for Dutch nursing homes (a total of 1872 working hours per year was assumed and extra payments for irregular shifts were not counted). This resulted in €0.22 per minute for a nursing assistant, €0.30 per minute for a nurse practitioner/wound care nurse, €0.34 per minute for a dietician/paramedic and €0.54 per minute for a nursing home physician. Time for a consultation of, for example, a nurse practitioner, dietician or other specialist was set at 15 minutes. And it was assumed that this consultation was valid for ten days; therefore each consultation time was divided by ten to arrive to a price per day. Costs for hiring/purchasing special anti-pressure ulcers mattresses were not counted, since it was not clear to what extent these mattresses are only used for therapy, or also for prevention or to what extent they are standard mattresses. Costs relating to nursing time for repositioning were counted as treatment costs, since the Dutch National Prevalence Surveys of Care Problems clearly show that repositioning is used far more in patients with than in patients without pressure ulcers (38% versus 13%) [19-21].

To estimate the mean duration of a sacral pressure ulcer literature sources [19-21,25-28] were used. However, no clear data were found on how long pressure ulcers in general persist; data in the literature varied from less than 2 weeks to 3 years. After discussion in the research project group a period of 16 days for a grade 1 and of 40 days for a grade 2 were set as the most plausible values for the duration.

The estimated time for repositioning was set at 10 minutes per turn, also based on data in the literature [7,29-34] that varied between 2 and 12.5 minutes per turn.

All calculations were performed in an Excel-spreadsheet.

### Model input values for investment costs

The investment costs were calculated with the following variables: purchase price of an AMS, the price of washing a single AMS, the durability/life span of an AMS, the required time for bringing the AMS to the laundry and back to the ward, and the frequency with which an AMS in bed is changed. Table 1 shows the input values for these variables. The main source was the Dutch trial, with supplementary data from other sources.

**Table 1:** Input values for investment costs for using the AMS

Input variables	Unit	Sources		Input values		
		Trial	Other	Low	Base	High
Purchase price AMS	€	85	15 quotes other AMS-suppliers:	75	85	95
			range 60-372;			
			median 87			
Price single washing	€	6-6.6	6 quotes other laundries:	4	6	8
			range 0.7-15;			
			median 2.5			
Frequency change AMS	days	4.8	Research protocol [37]: 3	1	3	5
Length resupply	days	3	-	1	3	5
Durability AMS	cycles	-	Research protocol [37]: 50	30	50	70
			Developer [40,41]: 60-100			
Needed stock	number	200	-	200	200	200
Needed extra stock for calamities	%	30	-	10	30	50

### Model input values for the savings from AMS use

The savings from the use of the AMS depend on the number of patients that are prevented from getting a sacral pressure ulcer grade 1 or 2 (effectiveness) multiplied by the costs for treatment of a sacral pressure ulcer grade 1 or 2, taking into account the ratio between grade 1 and 2 pressure ulcers.

Input data about the *effectiveness of the AMS *were based on the aforementioned Dutch RCT [13]. Since patients in this trial were followed from admission until 30 days later or until death or discharge, it means that the effectiveness ratio has a time frame of 30 days and it is assumed that no new pressure ulcers develop after this initial 30-day period. The incidence of sacral pressure ulcers in that trial was 14.7% in the control group versus 8.9% in the intervention group when analysed for all patients; it was 18.8% versus 11.7% in the group of patients with a risk on pressure ulcers and 24.8% versus 15.2% in the severely ADL-impaired patients. The differences in incidences from this trial were rounded to be used as input values for the effectiveness in the model (Table 2).

**Table 2:** Input values for the effectiveness of the AMS

Scenario	Input values for the difference in pressure ulcer incidence (%)		
	Low	Base	High
All patients	5	6	7
Patients at risk for pressure ulcers	6	7	8
Severely ADL-impaired patients	8	9	10

For the scenario using the AMS for all patients, an incidence difference of 6 was chosen as the base case value. Or in other words, use of the AMS for all admitted patients in the model nursing home with 468 admissions per year prevents 28 cases of sacral pressure ulcers. In the scenario where the AMS is used only for the 70% of patients with a pressure ulcer risk and a risk difference of 7, it prevents 23 cases of sacral pressure ulcers yearly. And in the scenario where the AMS is used only for the 45% of ADL-severely impaired patients and a risk difference of 9, the AMS prevents 19 cases of sacral pressure ulcers per year.

The *costs of treatment of sacral pressure ulcer *grade 1 or 2 were estimated on the basis of a combination of four approaches: the guideline approach, the National Prevalence Survey of Care Problems (NPSCP) approach, the empirical approach and the literature approach.

The first approach is based on the care described as appropriate in the Dutch national guideline on pressure ulcers [35]. The recommended actions in that guideline were seen as units and volume indicators and turned into cost-data (see Table 3). This approach resulted in estimated treatment cost of €14.16 per ulcer per day.

**Table 3:** Cost of treating pressure ulcers grade 1 or 2 per day, based on pressure ulcer guideline [35]

Action/Unit	Recommend volume	Price per unit	Formula	Costs per day (€)
Repositioning every 4 hours during a 24 hour period (by a care assistant)	6 × 10 minutes = 60 minutes	€0.22 per minute	60*0.22	13.20
Anti-pressure ulcer mattress	Not counted			Not counted
Informing patient	Not counted			Not counted
Assessing nutritional status and giving adequate nutrition	Single consultation with a dietician	€0.34 per minute	(15*0.34)/10	0.51
Assessing and treating pain & Assessing psychosocial situation	Single consultation with a nurse practitioner	€0.30 per minute	(15*0.30)/10	0.45
				
**TOTAL**				**14.16**

Secondly, data from The Dutch National Prevalence Survey of Care Problems were used http://www.lpz-um.eu[19-21]. In these surveys data about the prevalence, the location and the applied treatment of pressure ulcers are gathered yearly on a given day in all patients of participating health care institutions. We used data of three consecutive years (2006-2007-2008) on nursing home patients that were registered as having a sacral pressure ulcer (N = 2,772 patients from 157 different nursing homes, consisting of 1,517 patients with a grade 1, 820 grade 2, 288 grade 3 and 147 with a grade 4 sacral pressure ulcer). For these patients pressure ulcer treatment as recorded was converted into cost data. The treatment components consisted of repositioning, skin protection, wound care products, consultation with a dietician and other nursing activities. Because only categories of wound care products were recorded instead of a specific brand and size of product, the price for the cheapest wound care product from that category was used in the cost calculations. In case a recorded product is usually in situ for a number of days, the product price was divided by the usual number of days. For each patient we checked if such an activity or product was recorded for that day and if so, a price label was added. With regard to repositioning, these data mentioned only if the patients received repositioning but not the frequency or by whom the repositioning was performed. Therefore we assumed, based on the experiences of those members of the research group who were working in a nursing home, a repositioning frequency of six times daily, performed by a nursing assistant. Subsequently, a mean price for the patients with a pressure ulcer was calculated, resulting in €5.2 (sd = 6.37; min-max: 0-20.1) per day for a grade 1 and €6.3 (sd = 6.74; min-max: 0-19.6) for a grade 2 ulcer.

The third approach consisted of a convenience sample of nineteen nursing home patients from six different nursing homes that developed a sacral pressure ulcer. In these patients a diary was kept in which all therapy and the personnel involved in the treatment were prospectively charted. A wound-care nurse or pressure-ulcer nurse specialist filled out this diary every day from the onset of a sacral pressure ulcer during the whole period that it existed (or until discharge or death). She noted daily the grade of the ulcer, the wound care products used, special pressure relieving devices, kind and time of specialists that were consulted, diagnostic procedures and extra time for nursing personnel that was devoted to pressure ulcer care. This approach resulted in a mean treatment cost of €12.6 (sd = 4.4; min-max 3.7-20.6) per day for a grade 1 and €13.2 (sd = 5.1; min-max 3.5-18.5) for a grade 2 sacral pressure ulcer.

Finally, we made use of the articles related to the Dutch situation that were included in a recent systematic review [36] about treatment costs for sacral pressure ulcers in nursing home patients. However, the review included only one study relating to the Dutch situation. This Dutch cost-of illness study [5] gave a mean of €25 per patient per day for a grade 1 and €54 for a grade 2 pressure ulcer (values were discounted to year 2008).

An overview of the estimates found in the four approaches is shown in Table 4. Based on these different sources and after discussion in the research project group it was decided to use €15/day for a grade 1 and €17/day for a grade 2 pressure ulcer as input values for the base case in the model and +/-5€ for the sensitivity analyses. Expressed as costs per pressure ulcer period and with an assumed duration of 16 days for a grade 1 and 40 days for a grade 2 ulcer, this results in €240 and €680 respectively.

**Table 4:** Input values for mean cost of pressure ulcer treatment per day per grade (€)

	Sources				Input values		
	guideline	Npscp	empirical	literature	Low	Base	High
Grade 1	14.2	5.2	12.6	25.0	10	15	20
Grade 2	14.2	6.3	13.2	54.0	12	17	22

### Ethical considerations

For the randomized trial that was part of this economic analysis, patients were informed by the admitting nurse verbally and in written format about the research project before they were asked to participate and before they signed the informed consent form. At all times patients were free to withdraw from the study without having to give a reason for doing so.

The study protocol was approved by the certified Medical Ethics Review Board of the University Medical Centre of Utrecht (protocol number 06/287), on behalf of all the participating nursing homes.

The Board of Directors of each participating nursing home signed a form in which they stated they have read and understood the research protocol and that there were no obstacles at all to conducting the research in their organizations.

The scientific merits of the study protocol have been reviewed in the consecutive phases of the research funding process by independent reviewers of the funding organization ZonMw, the Netherlands Organization for Health Research and Development (http://www.zonmw.nl/en; ZonMw grant number 945-07-513).

Besides this, the research protocol, and more specifically the washing procedure of the sheepskins, has also been evaluated by the Dutch Working Party on Infection Prevention http://www.wip.nl/UK/ and approved as safe.

The protocol is registered in the Controlled Trial Register as ISRCTN17553857 and has been published earlier [37].

The patient data from The Dutch National Prevalence Survey of Care Problems http://www.lpz-um.eu were given to us without identifying information of both patients and institutions.

## Results

### What are the investment costs for using the AMS?

When the AMS is used for all newly admitted patients, it requires a yearly investment of nearly €94,000. When the AMS is used for selected groups only, the total investments costs are €65,578 for use solely in patients that are admitted with a risk on pressure ulcers and €42,158 for use in severely ADL- impaired patients only (Table 5). The washing accounts for almost 80% of the total costs.

**Table 5:** Annual investment costs for the AMS

	Unit	Scenario		
		All patients	Only patients with pressure ulcer risk	Only severely ADL-impaired patients
Initial investment for purchase taking into account the durability of the AMS	€	20,683	14,478	9,308
Costs for washing	€	73,000	51,100	32,850
				
**TOTAL costs per year**	€	**93,683**	**65,578**	**42,158**

### What are the savings from use of the AMS?

Savings are calculated from the number of patients in which sacral pressure ulcers are prevented, multiplied by the costs of sacral pressure ulcer treatment (Table 6).

**Table 6:** Annual savings from using the AMS

Scenario	N prevented from sacral pressure ulcer (70/30% grade 1/2)	Cost per period	Savings (€) per year
All patients	28	(28*70%) * 240 (28*30%) * 680	10,416
Only patients pressure ulcer risk	23	(23*70%) * 240 (23*30%) * 680	8,556
Only severely ADL-impaired patients	19	(19*70%) * 240 (19*30%) * 680	7,068

The total savings per year for the model nursing home are 10,416€, when the AMS is provided to all patients. If the model is changed to a scenario in which the AMS is only given to patients with a risk on pressure ulcers, the annual savings are 8,556€. In the scenario where the AMS is only given to severely ADL-impaired patients the savings are 7,068€ per year from prevented sacral pressure ulcer cases.

### What is the balance between the costs of and savings from using the AMS?

In the scenario where the AMS is used for all patients a yearly investment of €93,683 was needed and the savings were estimated at €10,416. This means an extra investment of €83,267 per year is required for the model nursing home of 100 beds and 468 new admissions annually. In other words, use of the AMS requires an additional investment of €178 per new patient. Translated into a price per patient per day, it means an extra investment of €2.28. This means that the net investment needed to prevent one case of sacral pressure ulcer by means of the AMS amounts €2,974 in this scenario. When the AMS is used more selectively, i.e. only for patients with a risk on pressure ulcers or only for severely ADL-impaired patients, then the total investment become smaller due to fewer patients, while the savings are relatively higher due to better effectiveness figures. In these scenarios the net investment drops to €2,479 and €1,847 respectively per case prevented for use in pressure ulcers-risk patients and severely ADL-impaired patients. Expressed as an additional cost per day per patient who gets an AMS, it means €2.23 and €2.14. Table 7 shows the figures for the 3 scenarios. In all scenarios the investment costs for use of the AMS are larger than the savings the AMS creates.

**Table 7:** Balance between investments for and savings from using the AMS

Scenario	Investment costs (€) per year	Savings (€) per year	Balance (€)	N prevented from sacral pressure ulcer	Costs to prevent 1 case of sacral pressure ulcer	Additional costs per day per patient
ALL patients	93,683	10,416	83,267	28	2,974	2.28
Only patients with pressure ulcer risk	65,578	8,556	57,022	23	2,479	2.23
Only severely ADL-impaired patients	42,158	7,068	35,090	19	1,847	2.14

### Sensitivity Analyses

Table 8 shows in decreasing order the relative impact of the various input variables as found by sensitivity analyses on the final balance, expressed as additional costs per day for use of the AMS in all patients. These sensitivity analyses show that the extra investment per day is largely influenced by the frequency of changing the AMS (due to the associated washing costs). Even if the low and high input values are altered to a changing frequency of every 4 days and every 2 days this factor remains the most influential. Second most influential factor is the cost of washing. Other factors such as the initial purchase price, the durability of the AMS, the effectiveness of the AMS or the costs of treating a pressure ulcer have much less impact on the balance. Even if the treatment costs per day are doubled, the balance keeps showing an extra investment of €2. In the scenarios where the AMS is selectively used, the same factors are the most influential in the sensitivity analyses.

**Table 8:** Sensitivity analyses for the additional costs per patient per day

Factor	Input values*			Additional cost per day			Difference between low-high, as % of base value
	Low	Base	High	Low	Base	High	
Frequency of changing	5	3	1	1.25	2.28	7.41	270
Cost of washing	4	6	8	1.61	2.28	2.95	59
Durability of AMS	70	50	30	2.12	2.28	2.66	24
Purchase price of AMS	75	85	95	2.21	2.28	2.35	6
Price per day of treating pressure ulcer grade 1	20	15	10	2.24	2.28	2.32	4
Price per day of treating pressure ulcer grade 2	22	17	12	2.23	2.28	2.33	4
Duration in days pressure ulcer grade 1	22	16	10	2.23	2.28	2.33	4
Duration in days pressure ulcer grade 2	50	40	30	2.24	2.28	2.32	4
Effectiveness of AMS	7	6	5	2.23	2.28	2.33	4
Ratio between grade 1 and 2 pressure ulcer	60/40	70/30	80/20	2.25	2.28	2.31	3
Length of stay	70	78	86	2.25	2.28	2.31	3
Extra stock	10	30	50	2.28	2.28	2.28	0
Length of wash/supply cycle	1	3	5	2.28	2.28	2.28	0

*The low input values are all these that influence the balance in the direction of favouring the AMS

## Discussion

This economic impact analysis of the AMS has demonstrated that the investment costs for using the AMS are larger than the savings obtained by avoided pressure ulcer treatment. The extra investment is approximately €2.28 per patient per day when used for all patients, decreasing to €2.23 when used only for pressure ulcers-risk patients and to €2.14 when used only for severely ADL-impaired patients. Expressed in another way, the costs in the respective scenarios are €2,974 to €2,479 and €1,847 to prevent one case of sacral pressure ulcer by means of the AMS. In practice these balance figures may vary, due to the large number of variables that influence them. Furthermore, there is much uncertainty in the model. The uncertainty pertains to all components of the model but most to the costs of treatment for pressure ulcers and less to the investment costs or the effectiveness rate. In particular, the costs per pressure ulcer period are surrounded by considerable uncertainty since these were based on a calculation of an uncertain price per day multiplied by an uncertain duration of a pressure ulcer.

It must, however, be stressed that conservative approaches have been used, meaning that input values for the model were never chosen in a way that clearly favoured the AMS. For instance, the costs of treatment for a pressure ulcer are more likely to be an underestimate. This is because not all possible treatment costs were counted, such as costs for special mattresses or the time involved for nurse assistants to go to and from a patient when repositioning; or the cheapest product of a category was chosen if it was not clear which product was used in reality and only direct costs were counted. However, although the treatment costs may be an underestimate, the sensitivity analyses show that even a doubling of these treatment costs doesn't result in a complete different balance.

This study has several limitations that have to be kept in mind when interpreting the results. First of all, the results presented are based on a model, which is by definition only a limited approximation of a real world situation. Furthermore the study is limited by the fact that we did not have data about qualitative savings for patients related to not having a sacral pressure ulcer. In this way QALY's (quality-adjusted life years) could not be computed and this hindered us in performing a complete cost-effectiveness analysis and in comparing our figures to accepted cost-effectiveness thresholds.

Moreover, a specific population with a majority of rehabilitation patients was used in the model. These rehabilitation patients have a shorter length of stay than the more chronic patients that usually form a large proportion of a normal real world nursing home. Besides, most rehabilitation patients get better, while the more chronic patients have a deteriorating course with an increasing risk on pressure ulcers. It may be the case that the balance would be more in favour of the AMS if it were used in such chronic patients.

The model also assumed that no pressure ulcers develop after the first 30 days following admission, and the AMS has no additional effect after this period. This is not certain and therefore the balance could in reality be more in favour of the AMS if the preventive effect lasts longer.

Sensitivity analyses demonstrated that the frequency of changing the AMS and the cost of washing are the most influencing factors. This means that the nursing home itself can influence the balance through negotiations with laundries about the price and through a well thought-out system of changing the AMS. It also means that there is a challenge for the industry to find laundry processes that are less expensive, in order to make the AMS more attractive.

From an economic point of view it would seem most logical to give the AMS only to a selection of patients, e.g. only to severely ADL-impaired patients, since the initial investment is lower due to the smaller number of patients, and the effectiveness of the AMS is relatively larger in these patients leading to a better balance between investments and savings. However, being selective also implies that a screening instrument is needed at admission, with all energy needed for that and with all possible mistakes/misclassifications that can occur. Therefore, from a practical point of view, it may be much easier to give the AMS to all patients initially and to reconsider the need for it later on.

In the debate whether to use the AMS or not, an evaluation of the extra investment is required. Is the extra 2.3€ per day to be valued as small or large? Expressed as percentage of the total amount of money a Dutch nursing home receives from the health care insurance for 1 day of care for a rehabilitation patient (ZZP-9 = €189.14; http://www.nza.nl[38]) the extra investment for using the AMS is about 1.2%. And who is at stake when the evaluations and decisions are made? Is it the manager, the physician, the specialized nurse, the nurse assistant or the patient? Furthermore, in that debate it has to be considered that the presented model was confined to monetary savings only. Qualitative savings such as gains in quality of life for patients that were prevented from pressure ulcers, comfort of the AMS for patients or practicality for nurses were not taken into account in this economic model. Although a recent study [39] gives figures on health utilities and the impact of pressure ulcers in long term care residents, these utilities were grouped into a utility for either a group with no or with grade 1 pressure ulcer and a group with grade 2, 3 and 4 together, making these figures not applicable to this study.

It is also not known yet how the additional investment for the AMS compares to extra investments for other alternative appliances/methods to prevent sacral pressure ulcers.

Accordingly, all these issues have to be taken into account before judging whether the AMS is good value for money or not.

At least it has now been proven in three RCTs [11-14] that the AMS is an effective aid for the prevention of pressure ulcers, and this paper provides an estimation of the required extra investments for use in a specific nursing home population. This is much more than can be said about other preventive aids for pressure ulcers.

Finally, this economic analysis was performed from the perspective of a Dutch nursing home. This means that these results can not be extrapolated in a straightforward way to other health care settings or to other countries. Extrapolating this model to another country would require data of that other country about salaries and costs of wound products, but it is unlikely that a completely different picture would arise. If the model were to be developed for a hospital setting, there is a chance that the balance would be more in favour of the AMS, because the costs of pressure ulcer treatment in a hospital setting will probably be larger due to extra length of stay for pressure ulcer patients or to higher salaries from the hospital personnel involved. This was demonstrated in a recent study [7], where treating a pressure ulcer grade 1 and 2 in a Dutch hospital setting involved a cost of respectively more than 30 or 50€ per day, which is much higher than the estimates of 15 and 17€ for a Dutch nursing home we applied in our analysis. Using another perspective than the nursing home, e.g. a societal perspective or a patient perspective, would also result in other outcomes.

## Conclusions

From this economic model study, it can be concluded that from the perspective of a nursing home, the investment costs for use of the Australian Medical Sheepskin in newly admitted rehabilitation patients are larger than the monetary savings obtained by preventing pressure ulcers.

Using the AMS involves an additional cost of approximately €2 per day per patient. By far the greatest influence on the balance between investment and savings is the cost of washing the AMS. However these conclusions are hampered by the considerable uncertainty about the costs of pressure ulcer treatment.

## Competing interests

The authors declare that they have no competing interests.

Neither the developer nor any retailer of Australian Medical Sheepskins or laundries contributed in any way to the funding of this study, and none of the authors have commercial or financial interests with these companies.

## Authors' contributions

All authors contributed substantially to the development of the study protocol and to the drafting of this paper. PM and AA performed all analyses; AFL, WA, RH, JH and HP commented all analyses. RH and HP had a substantial role in gathering data. PM, AA and AFL conceived the study and participated in its design and coordination. All authors read and approved the final manuscript.

## Pre-publication history

The pre-publication history for this paper can be accessed here:

http://www.biomedcentral.com/1472-6963/10/226/prepub
